# Efficacy and safety of progressively reducing biologic disease-modifying antirheumatic drugs in patients with rheumatoid arthritis in persistent remission: a study protocol for a non-inferiority randomized, controlled, single-blind trial

**DOI:** 10.1186/s13063-022-06543-y

**Published:** 2022-07-27

**Authors:** Shangwen Lei, Zijia Li, Xiaoli Zhang, Shuhong Zhou

**Affiliations:** 1 Department of Rheumatology, Gansu Provincial People’s Hospital, 204 Donggang West Road, Lanzhou, 730000 People’s Republic of China; 2 Department of Thoracic surgery, Gansu Provincial People’s Hospital, Lanzhou, 730000 People’s Republic of China

**Keywords:** Biologic disease-modifying antirheumatic drugs, Rheumatoid arthritis, Persistent remission, Trial

## Abstract

**Background:**

To compare the effects of two biologic disease-modifying antirheumatic drug (bDMARD) administration strategies on the maintenance effect and safety of patients with rheumatoid arthritis (RA) in remission, to analyze the effects of gradual drug reduction and dose maintenance treatment on clinical outcomes in patients who have achieved remission with different types of bDMARDs, to search and screen out people who may benefit from drug reduction strategies, and to provide references for drug reduction strategies and treatment options for patients with RA in remission, so as to help improve the safety of the treatment and reduce the economic burden.

**Methods:**

The study will be a 24-month non-inferiority randomized, controlled, single-blind trial and is planned to be launched in our hospital from September 2021 to August 2023. Patients will be randomized in a ratio of 2:1 to two groups: maintenance or injection spacing by 50%/gradual reduction of dosage every 3 months up to complete stop. When the patient relapses, return to the last effective dose. If the remission can be maintained, the medication of bDMARDs can be stopped 9 months after enrollment. The primary outcome will be the persistent flare rate.

**Discussion:**

Our study may provide a reference for the selection of drug reduction strategies and treatment options for patients with RA in remission, so as to help improve the safety of the treatment and reduce the economic burden.

**Trial registration:**

Chinese Clinical Trial Registry ChiCTR2100044751. Registered on 26 March 2021

## Background

Rheumatoid arthritis (RA) is a common systemic inflammatory autoimmune disease characterized by progressive inflammatory arthritis, joint swelling, and tenderness with a prevalence of 0.5–1% worldwide [1]. Over time, structural damage and weakened joint function may appear as evidenced by imaging progress [2, 3]. Severe cases not only lead to joint deformities and loss of function but may also be complicated by lung disease, cardiovascular disease, depression, and so on, which seriously affect the patient’s physical function and quality of life [1–4]. With the prolongation of the course of RA, the functional limitations and the incidence of disability in RA patients are also higher, causing serious economic and social burdens [5].

For a long time, the use of traditional disease-modified antirheumatic drugs (DMARDs), most commonly methotrexate in combination with glucocorticoids, has been the main therapy for RA [6]. With the understanding of the role of cytokines in autoimmune diseases, some targeted biological agents have gradually been synthesized and applied in treatment [7, 8]. The European Union Against Rheumatism (EULAR) first proposed the use of synthetic and biologic disease-modifying antirheumatic drugs (bDMARDs) to treat rheumatoid arthritis (RA) in 2010 [9]. A previous study reported that the remission rate of RA has increased significantly with the application of bDMARDs [10]. However, the use of bDMARDs puts the body in a long-term immunosuppressive state, which may increase the risk of infection and malignant tumors [11]. In addition, bDMARDs are relatively expensive, causing a heavy financial burden on patients and their families [12]. When patients get clinical remission, whether to reduce or even stop the use of bDMARDs becomes an important issue for clinical RA treatment.

Hence, the objectives of our study are (1) to compare the effects of two bDMARD administration strategies on the maintenance effect and safety of patients with RA in remission, (2) to analyze the effects of gradual drug reduction and dose maintenance treatment on clinical outcomes in patients who have achieved remission with different types of bDMARDs, (3) to search and screen out people who may benefit from drug reduction strategies, and (4) to provide references for drug reduction strategies and treatment options for patients with RA in remission, so as to help improve the safety of treatment and reduce the economic burden.

## Methods

### Study design

The trial is a randomized, controlled, single-blind design and is planned to be launched in our hospital from September 2021 to August 2023. A flow chart of the phases of the study is present in Table 1.

**Table 1:** Research flow chart

Interview							
Project	Screening period (enrollment to 3 ~ 0 days)	Follow-up					
		3 months ±10 day	6 months ±15 days	9 months ±22 days	12 months ±30 days	15 months ±37 days	18 months ±45 days
Fill in demographic information	√						
History-taking	√						
Vital signs tests	√						
Inclusion/exclusion criteria	√						
Informed consent signed	√						
Laboratory examination	√						
Imaging examination	√						√
Disease activity	√	√	√	√	√	√	√
Body functions	√	√	√	√	√	√	√
Life quality	√	√	√	√	√	√	√
Recurrence		√	√	√	√	√	√
Drug combination		√	√	√	√	√	√
Adverse event		√	√	√	√	√	√
Research summary							√

### Randomization procedures

Continuous RA patients in clinical remission will be collected and randomly divided into observation group and control group according to 2:1. The observation group will implement a progressively drug reduction strategy for bDMARDs guided by changes in disease activity (*n* = 156), and the control group will implement routine care and continue to use standard doses of bDMARDs (*n* = 78). Randomization will be generated by the computer, and the allocation will be hidden through opaque sealed envelopes in sequential numbers, that is, the treatment allocation corresponding to the serial number 001 to 234 (random coding table) will be listed, and the serial number corresponds to the subject number. The random code table should be kept by designated personnel. After the subjects are selected, the researcher will notify the custodian of the random code table of the corresponding subject number, and the latter will issue an instruction according to the random code table on whether the selected subject should enter the observation group or the control group. The researcher will have a corresponding record after receiving the instruction, and the corresponding allocation will be implemented in accordance with the instructions. Disease activity and X-rays will be evaluated by nurses and imaging doctors who are not aware of the group and provided to doctors treating RA. All nurses will receive uniform training and repeatedly joint assessment skills calibration to optimize reliability among raters.

### Participants and settings

#### Inclusion criteria

The following are the inclusion criteria:

1. Age ≥ 18 years old, no gender limit.
2. Patients with RA are diagnosed based on the RA classification criteria issued by the American College of Rheumatology (ACR) in 1987 [13] and the RA classification criteria issued by ACR/EULAR in 2010 [14].
3. Patients with a maintenance dosage of adalimumab, etanercept, rituximab, infliximab, and other bDMARDs in the treatment of RA in the past 6 months.
4. RA patients who maintained clinical remission in the past 6 months.
5. Patients with no structural damage to the hand-foot joint X-rays in the past 1 year.
6. The subjects voluntarily participate in the informed and signed informed consent form.

#### Exclusion criteria

The following are the exclusion criteria:

1. Patients who are currently receiving oral glucocorticoid therapy
2. RA patients who were received corticosteroids or short-term oral corticosteroids intra-articularly or parenterally in the past 6 months
3. Alcohol or drug addicts
4. Patients with mental disorders
5. Female patients who are pregnant, breastfeeding, or planning to become pregnant
6. Patients who have contraindications to maintenance treatment of bDMARDs
7. Patients with malignant tumors or those with a history of malignant tumors
8. Patients suffering from other autoimmune diseases
9. Patients who are or have participated in other clinical studies within 30 days

#### Drop-out criteria

The following are the drop-out criteria:

1. Patients with any changes or deaths that occurred during treatment or follow-up that were not related to study factors
2. There are emergency complications or other unexpected adverse events during the study
3. Patients who withdrew their informed consent and voluntarily asked to withdraw
4. Patient had a pregnancy event during the course of the clinical study
5. Any other circumstances under which the investigator considers the patient to be unsuitable for participation in the study

#### Interventions

Patients receiving conventional synthetic DMARDs (csDMARDs) combined with bDMARDs will continue the treatment of csDMARDs at a strict and stable dose throughout the research period.

#### Control group

In the control group, the patients will continue to maintain the standard injection therapy of the original dose of bDMARDs. The specific treatment plan was adalimumab 40 mg/14 days, etanercept 50 mg/7 days, and infliximab 3 mg/kg (maintenance every 8 ~ 12 weeks). If flare still occurs in the maintenance dose group during the research process, the rheumatologist immunologist will determine the intervention measures according to the condition, record the treatment in detail, and follow up to the end point.

#### Observation group

The drug reduction strategy will be achieved by bDMARD injection interval, which relies on the disease activity assessed by DAS28 every 3 months to gradually increase the interval of subcutaneous injections or reduce the injection dose. All other treatments will maintain a stable dose throughout the trial. The specific drug reduction strategies are adjust according to the DAS28 score at the time of enrollment and every 3 months thereafter, increase the interval of the original drug regimen at enrollment, and when there is no flare during the 3-month follow-up (baseline comparison ∆DAS28 > 1.2 or ∆DAS28 > 0.6 and current DAS28 ≥ 3.2), then continue to reduce the drug. When the patient relapses, return to the last effective dose. The time interval is increased by about 50% each time, among which infliximab is gradually reduced due to its different dosage and cycle, and the injection dose is reduced by about 1/3 each time without changing the injection interval.

If the remission can be sustained, the medication of bDMARDs should be stopped 9 months after enrollment. The injection intervals of each bDMARDs will be as follows:

Adalimumab: 40 mg/14 days→40 mg/21 days→40 mg/28 days→40 mg/42 days→drug withdrawal.

Etanercept: 50 mg/7 days→50 mg/10 days→50 mg/14 days→50 mg/21 days→drug withdrawal.

All patients will be told that when symptoms related to flare occur, such as joint swelling and pain, they should see a doctor within 2–7 days. Intra-articular steroids and/or short courses of non-steroidal anti-inflammatory drugs (up to 14 days) can be used to treat relapses. For patients with persistent relapses who are still ineffective after increasing the dose, they should be treated in accordance with clinical guidelines, for example, switch to another bDMARD. All patients with persistent relapse will no longer try to reduce the drug strategy.

#### Drug accountability

The patient’s medications in the past 6 months, including the types and dosage of csDMARDs (methotrexate, sulfasalazine, hydroxychloroquine, leflunomide, etc.) and bDMARDs (adalimumab, etanercept, rituximab, tocilizumab, etc.), will be recorded in detail.

### Primary outcomes

The main outcome of this study is the persistent flare rate.

Flare: Compared with baseline, if ∆DAS28 > 1.2 or ∆DAS28 > 0.6 and current DAS28 ≥ 3.2, it means that the patient has relapsed.

Persistent flare: When the flare lasts for 3 months, it is defined as a persistent flare [15, 16].

Observation time point: During the follow-up period, assess disease activity at least every 3 months.

### Secondary outcomes

The secondary outcomes include the cumulative flare rate, disease activity during follow-up, physical function, quality of life, imaging performance and imaging progress, the proportion of clinical remission and low disease activity (LDA) maintained at the end of the follow-up, the proportion of patients in the observation group who successfully reduced the drug, and the proportion of patients in observation group who successfully discontinued the drug, occurrence of adverse events, et al.

Disease Activity Score in 28 joints (DAS28) [17, 18] will be used to judge disease activity. DAS28 ≤ 2.6 indicates clinical remission, 2.6 < DAS28 ≤ 3.2 indicates LDA, 3.2 < DAS28 ≤ 5.1 indicates moderate disease activity, and DAS28 > 5.1 indicates high disease activity. DAS28 is evaluated from four aspects: the number of tender joints (TEN28), the number of swollen joints (SW28), the erythrocyte sedimentation rate (ESR), and the overall health (OH) assessed by the patients. The specific calculation formula is: DAS28 = 0.56 × $\sqrt{\mathrm{TEN}28}$ + 0.28 × $\sqrt{\mathrm{SW}28}$ + 0.7 × lnESR + 0.014 × OH.

Disease characteristics include laboratory examinations such as rheumatoid factor (RF), anti-citrullinated protein antibody (ACPA), ESR, and C-reactive protein (CRP).

For the imaging performance, the hands and wrist joints will be examined by X-rays. Two experienced imaging doctors will use van der Heijde Modified Sharp Score (vdHSS) [19, 20] to evaluate the structural damage from two aspects of bone erosion and bone joint space stenosis.

Imaging progression will be evaluated by the difference between the two vdHSS (∆vdHSS) after treatment and at baseline, ∆vdHSS > 0.5.

Physical function was assessed using the Health Assessment Questionnaire Disability Index (HAQ-DI), with a higher score indicating poorer functioning [21].

The European Quality of Life Five Dimensions Five Level (EQ-5D-5L) was used to evaluate the quality of life of patients, mainly from five dimensions: mobility, self-care ability, daily activity ability, pain or discomfort, and anxiety or depression [15].

### Clinical and laboratory assessment

The clinical and laboratory assessments performed during the trial are shown in Table 1.

### Statistical methods

#### Sample size

The main outcome of this trial is the persistent flare rate of the two groups of patients. With reference to published studies, it is assumed that the sustained flare rate of the control group is 14% and that of the observation group is 17%. The sample size calculation was based on a one-sided test with *α* = 0.025, *β* = 0.1, and a non-inferiority margin of 20%. For ethical reasons that more subjects benefit from any possible positive outcomes of this treatment, eligible patients will undergo randomization at a 2:1 ratio (*r* = 0.5). Using PASS for sample size calculation, the estimated sample size needed to be 140 for the observation group and 70 for the control group to achieve a power of 90%. Considering the dropout rate and the missing data, the final sample size required will be 234 cases, 156 cases in the observation group, and 78 cases in the control group.

### Statistical analysis

All data analyses will be carried out according to a pre-established statistical analysis plan. The analysis will be conducted on the basis of the intention-to-treat (ITT) and the per-protocol set (PPS), because the ITT analysis may underestimate the difference between the different treatment groups in the non-inferiority study, leading to false positive studies. Safety analysis will be performed on the data with laboratory inspection data, adverse events, and adverse reaction data. The number and reasons of patients excluded and withdrawn will be reported to ensure internal validity. Descriptive analysis will be used to describe missing data on determinants/covariates, and the mechanism of missing data will be studied. When the assumption of (completely) missing at random is met, multiple imputation will be used to estimate missing values to improve accuracy and reduce bias. All statistical analyses will be performed using the SPSS 24.0 software. The description of quantitative indicators will calculate the mean, standard deviation, median, and minimum and maximum values. Normally distributed measurement data are expressed as mean ± standard deviation. Counting data are expressed in terms of number of cases and composition ratio (*N* (%)). The group *F* test, Wilcoxon rank sum test, or *χ*^2^ test will be used to compare the demographic data and other baseline value indicators to measure the balance between the groups. The *t* test and the Wilcoxon rank sum test will be used to compare the differences between the measurement data. For the count data, the *χ*^2^ test or Fisher’s exact probability method will be used for comparison. DAS28, HAQ-DI, and EQ-5D-5L will adopt the analysis method of multivariate test. In order to assess whether the drug reduction strategy would lead to relapse, a univariate Cox proportional hazard model will be used to compare the relapse rate between treatment groups. The Kaplan-Meier method will be used to construct the cumulative flare rate. Univariate and multivariate Cox regression will be used to analyze the influencing factors of recurrence. All statistical tests will use two-sided tests, *P* ≤ 0.05 will be considered as statistically significant differences in the test.

#### Data collection

During the clinical research process, the data of the subjects will be collected on the original data, and the researcher will use EDC for data collection. The researcher will complete and truthfully record according to the requirements of the clinical research protocol. For data that significantly deviates from the clinically acceptable range, it must be verified, and necessary explanations should be made. After the completed EDC is checked, it will be transferred to data management personnel for data entry, management, and statistics. After the handover, the content of EDC will no longer be modified.

#### Adverse events

Adverse events refer to unfavorable medical events that occurred during the clinical research process, whether they are related to the research or not, they should be recorded on the EDC Adverse Event Table. The researcher evaluates the adverse events observed or stated by the subjects, including the date of occurrence, duration, nature, examinations done, severity, and outcome. For each new adverse event or recurring adverse event, a new form must be filled in. In the event of an adverse event or even a serious adverse event during the clinical research process, the researcher should immediately take appropriate treatment measures to the subject and report in writing to the ethical institution of the hospital to which it belongs.

#### Data registration and monitoring

Each selected case must be filled out by the investigator and completed the EDC applet. After verification, the relevant personnel (data management personnel) will be transferred to the data entry, management, and statistical work. For the questions in the EDC applet, the data manager will generate a Question Answer Form and send an inquiry to the researcher. The researcher should answer and return as soon as possible. The data manager will modify the data, confirm, and enter the data according to the researcher’s answer. If necessary, the Question Answer Form can be issued again.

#### Ethics and informed consent

Clinical research must follow the Declaration of Helsinki and relevant clinical research norms and regulations in China. Before the start of the clinical research, the clinical research can be implemented only after the Medical Ethics Committee of Gansu Provincial People’s Hospital approves the clinical research plan. Before subjects are selected for this study, the investigator will give the subject a detailed introduction of the background, purpose, procedures, risks and other questions of the clinical study, and answer the research-related questions raised by the subjects. Informed consent for the trial will be obtained from all patients voluntarily before study enrolment by the investigators.

## Discussion

The 2019 update of the EULAR RA management recommendations addressed that the ongoing development of bDMARD or targeted synthetic DMARDs (tsDMARD) has allowed for an increasing proportion of patients to attain the treatment target [9]. However, the use of bDMARDs will make the body in a long-term immunosuppressive state and will increase the risk of infection and malignant tumors [12]. The past experience of using csDMARDs suggests that it should be cautious when deciding to stop treatment. Stop treatment may lead to flare of the disease, and it may be more difficult to obtain remission again [21, 22].

Several trials demonstrated that the proportion of patients who maintain clinical remission after stopping bDMARDs directly, especially for long-term or even refractory RA, the flare rate may be as high as 75% [23, 24]. A phase III study trial of certuzumab showed that there was no significant difference in the outcome of continuous standard dose and increased injection interval for patients with long-term clinical remission of RA, but both of the two outcomes are superior to those who directly stop the administration of certuzumab [25]. A meta-analysis conducted by Henaux et al. indicated that discontinuing bDMARDs increases the risk of loss of remission or LDA and imaging progression, while reducing the dose of bDMARDs increases the risk of loss of remission, but does not increase the risk of flare or imaging progression [26]. The dose reduction strategy of subcutaneous tumor necrosis factors (TNF) inhibitors trail showed that the dose reduction strategy guided by disease activity produces results that are equivalent to the maintenance of the original dose [27]. Compared with the conventional care group, the disease activity-guided drug reduction group had a similar proportion of patients who relapsed at 18 months (12% vs 10%). In the drug reduction group, 20% of the patients successfully stopped the TNF inhibitor treatment, 43% of the patients successfully increased the injection interval, and the remaining 37% of the patients could not achieve drug reduction. There were no differences in functional status, quality of life, and related imaging progress between the two groups. Long-term follow-up found that the effectiveness and safety of this drug reduction strategy in RA can be maintained for 3 years, which greatly reduces the use of TNF inhibitors and improves cost-effectiveness.

In a word, it is essential to determine the best strategy for reducing the dose of bDMARDs or even stopping the treatment to improve the safety of the treatment and control the cost of treatment after remission of RA. At present, most of the existing studies focused on the reduction and withdrawal strategies of tumor necrosis factors (TNF) inhibitors, and few of them reported other types of biological agents. In account of this, our study intends to enroll RA patients who have obtained clinical remission with different bDMARDs, and then implement bDMARD gradual drug reduction and dose maintenance treatment after randomization to compare the maintenance effects of two bDMARD administration strategies on patients with RA in remission. Our study may provide a reference for the selection of drug reduction strategies and treatment options for patients with RA in remission, so as to help improve the safety of the treatment and reduce the economic burden.

### Trial status

Recruitment started in May 2021, and the trial is still recruiting.

## Data Availability

Not applicable.

## Abbreviations

DMARDs: Disease-modified antirheumatic drugs
bDMARDs: Biologic disease-modifying antirheumatic drugs
RA: Rheumatoid arthritis
ACR: American College of Rheumatology
EULAR: European Union Against Rheumatism
csDMARDs: Conventional synthetic DMARDs
LDA: Low disease activity
DAS28: Disease Activity Score in 28 Joints
TEN28: Number of tender joints
SW28: Number of swollen joints
ESR: Erythrocyte sedimentation rate
OH: Overall health
RF: Rheumatoid factor
ACPA: Anti-citrullinated protein antibody
CRP: C-reactive protein
vdHSS: van der Heijde Modified Sharp Score
HAQ-DI: Health Assessment Questionnaire Disability Index
EQ-5D-5L: European Quality of Life Five Dimensions Five Level
ITT: Intention-to-treat
PPS: Per-protocol set
tsDMARD: Targeted synthetic DMARDs
TNF: Tumor necrosis factors

## References

[1] Sparks JA (2019). Rheumatoid arthritis. Ann Intern Med.

[2] Escalante A, Haas RW, del Rincon I (2005). A model of impairment and functional limitation in rheumatoid arthritis. BMC Musculoskelet Disord.

[3] Hulsmans HM, Jacobs JW, van der Heijde DM, van Albada-Kuipers GA, Schenk Y, Bijlsma JW (2000). The course of radiologic damage during the first six years of rheumatoid arthritis. Arthritis Rheum.

[4] Aletaha D, Smolen JS (2018). Diagnosis and management of rheumatoid arthritis: a review. JAMA..

[5] Cooper NJ (2000). Economic burden of rheumatoid arthritis: a systematic review. Rheumatology (Oxford).

[6] Singh JA, Saag KG, Bridges SL, Jr77, et al. 2015 American College of Rheumatology Guideline for the treatment of rheumatoid arthritis. Arthritis Rheum 2016;68:1–26. 10.1002/art.39480 26545940

[7] McInnes IB, Schett G (2011). The pathogenesis of rheumatoid arthritis. N Engl J Med.

[8] McInnes IB, Buckley CD, Isaacs JD (2016). Cytokines in rheumatoid arthritis - shaping the immunological landscape. Nat Rev Rheumatol.

[9] Smolen JS, Landewé R, Breedveld FC (2010). EULAR recommendations for the management of rheumatoid arthritis with synthetic and biological disease-modifying antirheumatic drugs. Ann Rheum Dis.

[10] Aga AB, Lie E, Uhlig T (2015). Time trends in disease activity, response and remission rates in rheumatoid arthritis during the past decade: results from the NOR-DMARD study 2000-2010. Ann Rheum Dis.

[11] Fautrel B, Den Broeder AA (2015). De-intensifying treatment in established rheumatoid arthritis (RA): why, how, when and in whom can DMARDs be tapered?. Best Pract Res Clin Rheumatol.

[12] Haschka J, Englbrecht M, Hueber AJ (2016). Relapse rates in patients with rheumatoid arthritis in stable remission tapering or stopping antirheumatic therapy: interim results from the prospective randomised controlled RETRO study. Ann Rheum Dis.

[13] Arnett FC, Edworthy SM, Bloch DA (1988). The American rheumatism association 1987 revised criteria for the classification of rheumatoid arthritis. Arthritis Rheum.

[14] Aletaha D, Neogi T, Silman AJ (2010). 2010 rheumatoid arthritis classification criteria: an American College of Rheumatology/European league against rheumatism collaborative initiative. Ann Rheum Dis.

[15] Bouman CA, van Herwaarden N, van den Hoogen FH (2017). Long-term outcomes after disease activity-guided dose reduction of TNF inhibition in rheumatoid arthritis: 3-year data of the DRESS study - a randomised controlled pragmatic non-inferiority strategy trial. Ann Rheum Dis.

[16] van der Maas A, Lie E, Christensen R (2013). Construct and criterion validity of several proposed DAS28-based rheumatoid arthritis flare criteria: an OMERACT cohort validation study. Ann Rheum Dis.

[17] Burmester GR, Buttgereit F, Bernasconi C (2020). Continuing versus tapering glucocorticoids after achievement of low disease activity or remission in rheumatoid arthritis (SEMIRA): a double-blind, multicentre, randomised controlled trial. Lancet.

[18] van Gestel AM, Haagsma CJ, van Riel PL (1998). Validation of rheumatoid arthritis improvement criteria that include simplified joint counts. Arthritis Rheum.

[19] van der Heijde D (2000). How to read radiographs according to the sharp/van der Heijde method. J Rheumatol.

[20] Fautrel B, Pham T, Alfaiate T (2016). Step-down strategy of spacing TNF-blocker injections for established rheumatoid arthritis in remission: results of the multicentre non-inferiority randomised open-label controlled trial (STRASS: spacing of TNF-blocker injections in rheumatoid ArthritiS study). Ann Rheum Dis.

[21] Fries JF, Spitz PW, Young DY (1982). The dimensions of health outcomes: the health assessment questionnaire, disability and pain scales. J Rheumatol.

[22] ten Wolde S, Breedveld FC, Hermans J (1996). Randomised placebo-controlled study of stopping second-line drugs in rheumatoid arthritis. Lancet..

[23] Smolen JS, Breedveld FC, Burmester GR (2016). Treating rheumatoid arthritis to target: 2014 update of the recommendations of an international task force. Ann Rheum Dis.

[24] Brocq O, Millasseau E, Albert C (2009). Effect of discontinuing TNFalpha antagonist therapy in patients with remission of rheumatoid arthritis. Joint Bone Spine.

[25] Smolen JS, Emery P, Ferraccioli GF (2015). Certolizumab pegol in rheumatoid arthritis patients with low to moderate activity: the CERTAIN double-blind, randomised, placebo-controlled trial. Ann Rheum Dis.

[26] Weinblatt ME, Bingham CO, Burmester GR (2017). A phase III study evaluating continuation, tapering, and withdrawal of certolizumab pegol after one year of therapy in patients with early rheumatoid arthritis. Arthritis Rheum.

[27] Henaux S, Ruyssen-Witrand A, Cantagrel A (2018). Risk of losing remission, low disease activity or radiographic progression in case of bDMARD discontinuation or tapering in rheumatoid arthritis: systematic analysis of the literature and meta-analysis. Ann Rheum Dis.

